# P-Glycoprotein Acts as an Immunomodulator during Neuroinflammation

**DOI:** 10.1371/journal.pone.0008212

**Published:** 2009-12-08

**Authors:** Gijs Kooij, Ronald Backer, Jasper J. Koning, Arie Reijerkerk, Jack van Horssen, Susanne M. A. van der Pol, Joost Drexhage, Alfred Schinkel, Christine D. Dijkstra, Joke M. M. den Haan, Teunis B. H. Geijtenbeek, Helga E. de Vries

**Affiliations:** 1 Department of Molecular Cell Biology and Immunology, VU University Medical Center, Amsterdam, The Netherlands; 2 Department of Molecular Biology, Netherlands Cancer Institute (NKI), Amsterdam, The Netherlands; 3 Center for Experimental and Molecular Medicine, Academic Medical Center, Amsterdam, The Netherlands; Centre de Recherche Public de la Santé (CRP-Santé), Luxembourg

## Abstract

**Background:**

Multiple sclerosis is an inflammatory demyelinating disease of the central nervous system in which autoreactive myelin-specific T cells cause extensive tissue damage, resulting in neurological deficits. In the disease process, T cells are primed in the periphery by antigen presenting dendritic cells (DCs). DCs are considered to be crucial regulators of specific immune responses and molecules or proteins that regulate DC function are therefore under extensive investigation. We here investigated the potential immunomodulatory capacity of the ATP binding cassette transporter P-glycoprotein (P-gp). P-gp generally drives cellular efflux of a variety of compounds and is thought to be involved in excretion of inflammatory agents from immune cells, like DCs. So far, the immunomodulatory role of these ABC transporters is unknown.

**Methods and Findings:**

Here we demonstrate that P-gp acts as a key modulator of adaptive immunity during an *in vivo* model for neuroinflammation. The function of the DC is severely impaired in P-gp knockout mice (Mdr1a/1b−/−), since both DC maturation and T cell stimulatory capacity is significantly decreased. Consequently, Mdr1a/1b −/− mice develop decreased clinical signs of experimental autoimmune encephalomyelitis (EAE), an animal model for multiple sclerosis. Reduced clinical signs coincided with impaired T cell responses and T cell-specific brain inflammation. We here describe the underlying molecular mechanism and demonstrate that P-gp is crucial for the secretion of pro-inflammatory cytokines such as TNF-α and IFN-γ. Importantly, the defect in DC function can be restored by exogenous addition of these cytokines.

**Conclusions:**

Our data demonstrate that P-gp downmodulates DC function through the regulation of pro-inflammatory cytokine secretion, resulting in an impaired immune response. Taken together, our work highlights a new physiological role for P-gp as an immunomodulatory molecule and reveals a possible new target for immunotherapy.

## Introduction

Multiple sclerosis (MS) is the most common chronic inflammatory disease of the central nervous system (CNS), characterized by the presence of demyelinated lesions throughout the brain [1]–[4]. Experimental autoimmune encephalomyelitis (EAE) is a widely accepted animal model for MS, sharing its clinical, immunological and pathological characteristics [5]. The mechanisms of CNS inflammation in MS and EAE involve generation of autoreactive, myelin specific T helper (Th) cells in the peripheral lymphoid organs, which subsequently enter the brain, initiate an immune response and eventually cause destruction of myelin sheaths and axonal loss [6]. Antigen-presenting cells like dendritic cells (DCs) are important regulators of immune responses by presenting their captured antigens to specific T cells [7]. In general, the maturation status of DCs is a key determinant of how the immune response will evolve [8], [9]. Molecules or proteins that regulate DC maturation and thereby control immune responses are under extensive investigation, since this may provide tools for immune modulation.

One possible candidate for immunomodulation is P-glycoprotein (P-gp; ABCB1), a well-known multi-drug resistance (MDR) efflux pump, which transports a variety of substrates and drugs through the membrane against a concentration gradient at the cost of ATP hydrolysis [10], [11]. P-gp was originally discovered as a prototypic transporter involved in MDR of tumor cells [12], and was the first drug efflux transporter to be detected on blood-brain barrier endothelial cells [13]. P-gp is also expressed on a variety of immune cells like monocytes, DCs, T and B cells [14] and is involved in the efflux of inflammatory molecules such as steroids, prostaglandins and cytokines [14]–[19], suggesting a function in immunomodulation. A limited number of studies have implied that P-gp can modulate immune responses by regulating the emigration of Langerhans cells to lymphoid organs [20] and APC maturation in vitro via IL-12 secretion [21], but *in vivo* relevance is lacking.

Despite the reports suggesting immune-related functions of P-gp, data on how P-gp exerts its action during immune responses remains unknown. Therefore, the goal of our study was to investigate the potential immunomodulatory role of P-gp *in vivo*. We here demonstrate that DCs from P-gp knockout mice (Mdr1a/1b−/−, [22]) are severely impaired in their maturation and T cell stimulatory capacity. Consequently, Mdr1a/1b−/− mice displayed reduced clinical signs of EAE, which coincided with decreased inflammation in the brain and an overall reduced T cell response. Taken together, our findings highlight a novel immunomodulatory role for P-gp, which may open new therapeutic avenues to interfere in the pathogenesis of (auto)immune-related or inflammatory diseases.

## Results

### Mdr1a/1b −/− Mice Have Reduced Clinical Signs of EAE

**Figure 1:**
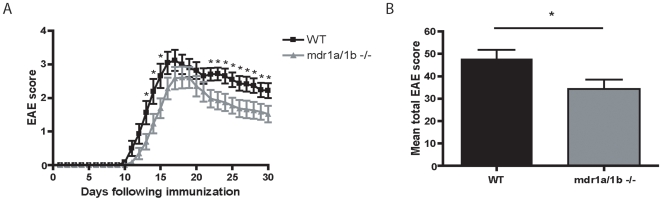
Reduced clinical signs in *Mdr1a/1b−/−* mice during acute and progressive phase of EAE. (A) Clinical signs of EAE induced by immunization of wild-type (WT) and Mdr1a/1b−/− mice with rMOG (1–125) showing mean clinical scores (+/− SEM) of two independent experiments (*p<0.002 Mann-Whitney, n = 23 mice per group). (B) Mean total EAE score per WT or Mdr1a/1b −/− mouse. *p<0.05.

**Figure 2 pone-0008212-g002:**
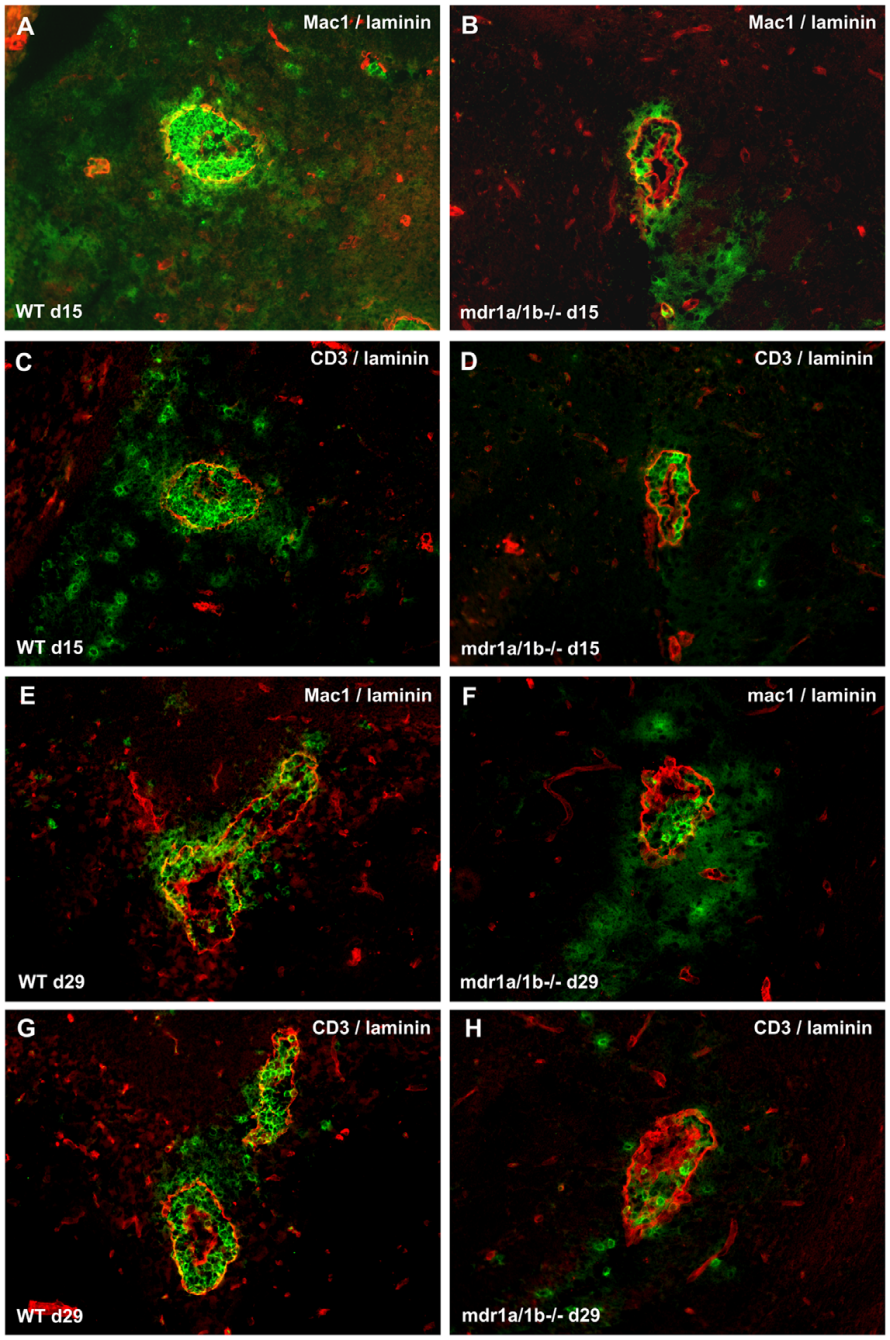
Decreased brain inflammation in *Mdr1a/1b−/−* EAE lesions. Brains were isolated from EAE mice on day 15 (A–D) and day 29 (E–H) after immunization and the cerebellum white matter was analyzed for the infiltration of CD45^+^ mac1^+^ macrophages (green; A,B,E,F) or CD45^+^ CD3^+^ T cells (green; C,D,G,H) and laminin positive (red) basement membranes around vessels in WT (left panel) or Mdr1a/1b−/− (right panel) mice. Images represent representative tissues from 4 mice per group. Magnification 200×.

EAE was induced in Mdr1a/1b−/− and wild-type mice using recombinant myelin oligodendrocyte glycoprotein (rMOG). Notably, Mdr1a/1b −/− mice showed significantly reduced clinical signs of disease (*p<0.05) compared to wild-type animals during the acute (day 15) and progressive (day 29) phase of disease (Figure 1a, Table 1). Moreover, the total EAE score per animal was significantly lower in Mdr1a/1b−/− mice compared to wild-type mice (Figure 1b). The observed differences in clinical signs were associated with decreased demyelination in the brain of Mdr1a/1b−/− EAE animals (Figure S1). Decreased demyelination in Mdr1a/1b−/− animals coincided with diminished brain inflammation as determined by the reduced numbers of infiltrated macrophages in EAE lesions during the acute (Figure 2a,b) and progressive (Figure 2e,f) phase of disease (see Table S3 for a detailed overview). Notably, almost no CD3^+^ T cell infiltrates were observed in Mdr1a/1b −/− EAE lesions during the acute (Figure 2c,d) and progressive (Figure 2g,h) phase of disease (Table S3), suggesting that the immune response and in particular the specific T cell response is selectively affected in Mdr1a/1b −/− mice during EAE.

**Table 1:** Clinical characteristics of EAE in WT and mdr1a/1b −/− mice.

Mice (n)	Incidence	Day of onset (+/− SD)	Survival	Maximal score (+/− SEM)	Maximal score day 29 (+/−SEM)
WT (23)	23/23	13.0 (+/−0.4)	22/23	3.1 (+/−0.06)	2.2 (+/−0.04)
Mdr1a/1b −/− (23)	21/23	13.8 (+/−0.3)	23/23	2.6 (+/−0.07)	1.6 (+/−0.05)*

* p<0.05 compared to WT mice.

### Reduced Th1 and Th2 Response in Mdr1a/1b−/− Mice during EAE

**Figure 3:**
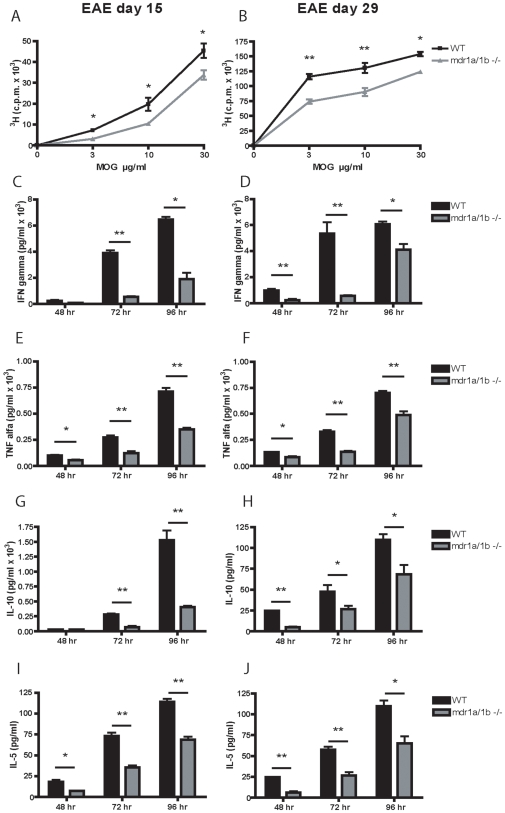
Impaired Th1 and Th2 response in *Mdr1a/1b−/−* mice during EAE. T cell proliferation in lymph node cells isolated from wild-type (WT) and Mdr1a/1b−/− mice was assessed upon re-stimulation with different concentrations of rMOG (1–125) on day 15 (A) or day 29 (B) of EAE. Supernatants from T cell proliferation assays upon re-stimulation with 10 µg/ml rMOG (1–125) were harvested and the production of the Th1 cytokines IFN-γ (C,D) or TNF-α (E,F) and the Th2 cytokines IL-10 (G,H) or IL-5 (I,J) was measured on different time points (48, 72 and 96 hr). Experiments were performed in triplicate using 4 mice per group and were presented as the mean +/− SEM. *p<0.05, **p<0.01.

To determine whether T cell responses to rMOG were reduced in Mdr1a/1b−/− mice, we compared the proliferative capacity and cytokine secretion of lymph node cells from Mdr1a/1b−/− and wild-type animals at different time points of EAE. Interestingly, lymph node cells from rMOG-immunized Mdr1a/1b−/− mice showed significantly impaired MOG specific T cell proliferation during the acute and progressive phase of disease compared to wild-type animals (Figure 3a,b). Furthermore, MOG specific secretion of Th1 cytokines IFN-γ and TNF-α (Figure 3c–f) and Th2 cytokines IL-10 and IL-5 (Figure 3g–j) by lymph node cells was significantly decreased in rMOG-immunized Mdr1a/1b−/− mice compared to wild-type animals, whereas no changes were observed in the secretion of the Th17 cytokine IL-17 (data not shown). These results demonstrate a decreased Th1 and Th2 response in Mdr1a/1b−/− mice compared to wild-type animals upon immunisation with rMOG.

### P-gp Regulates Inflammatory Cytokine Extrusion but Not Polyclonal T Cell Activation

**Figure 4:**
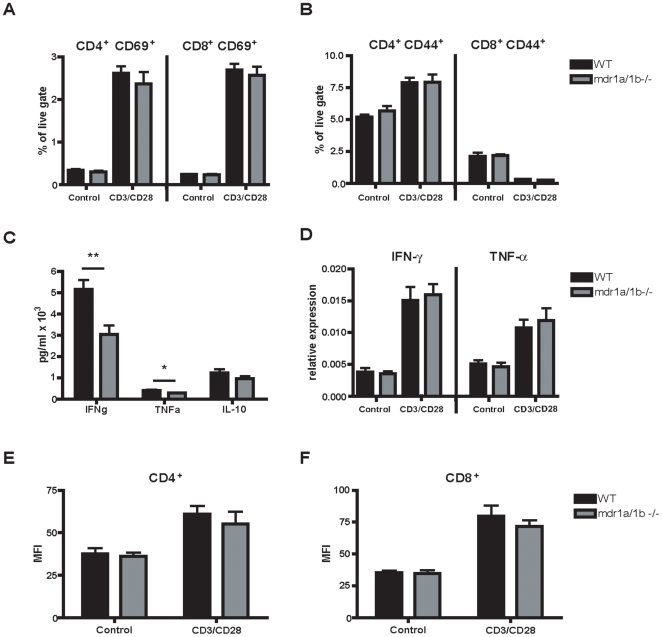
P-gp does not affect polyclonal T cell activation. Lymph node cells isolated from WT and Mdr1a/1b−/− mice were stimulated with anti-CD3/anti-CD28 for 5 hr and subsequently stained for T cell activation markers CD69 (A) or CD44 (B) on live cell gated CD4^+^ or CD8^+^ T cells. After anti-CD3/anti-CD28 stimulation, cytokine production of IFN-γ, TNF-α or IL-10 was measured (C) and IFN-γ and TNF-α transcripts were detected by RT-PCR and presented as relative expression compared to GAPDH (D). Intracellular IFN-γ production in CD4^+^ (E) or CD8^+^ T cells (F) was determined on permeabilized lymph node cells after anti-CD3/anti-CD28 stimulation. Experiments were performed in triplicate using 5 mice per group and were presented as the mean +/− SEM. *p<0.05, **p<0.01.

Reduced brain infiltration of CD3^+^ T cells in Mdr1a/1b−/− EAE lesions (Figure 2) and a reduced T cell response to rMOG in Mdr1a/1b−/− mice suggests that the activation status of these T cells is impaired. To test this, we assessed the activation capacity of CD4^+^ and CD8^+^ T cells upon stimulation with anti-CD3/anti-CD28 [23]. No differences in the expression of T cell activation markers like CD44 and CD69 (Figure 4a,b) or CD25 and CD62L (data not shown) were observed between CD4^+^ and CD8^+^ T cells from Mdr1a/1b−/− and wild-type mice after anti-CD3/anti-CD28 stimulation. However, anti-CD3/anti-CD28 stimulation of Mdr1a/1b −/− lymph node cells resulted in a reduced release of the inflammatory cytokines TNF-α and IFN-γ compared to wild-type cells (Figure 4c). In contrast, no differences were observed in the production of IFN-γ or TNF-α transcripts after anti-CD3/anti-CD28 stimulation between wild-type and Mdr1a/1b−/− lymph node cells (Figure 4d), which correlated with no differences in intracellular IFN-γ levels in CD4^+^ T cells (Figure 4e) or CD8^+^ T cells (Figure 4f). Together, these results indicate that P-gp is involved in the cellular extrusion of inflammatory cytokines from lymph node cells but not its cellular production. Moreover, P-gp does not affect the ability of T cells to become activated, pointing to a distinct mechanism during EAE pathology. As DCs are crucial mediators of immune responses by regulating specific T cell responses [7], P-gp may perform its immunomodulatory function predominantly on DCs during DC-induced T cell responses.

### Specific P-gp Blockade Inhibits T Cell Proliferation during DC-T Cell Interactions

**Figure 5:**
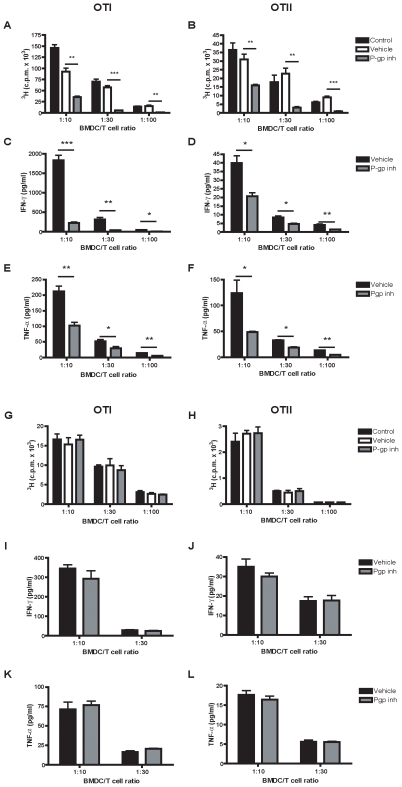
P-gp is necessary for CD4^+^ and CD8^+^ T cell proliferation. Bone marrow derived DCs (BMDCs) were coated with MHC class I OVA_257–264_ peptide or MHC class II OVA_323–339_ peptide and were used as stimulators for T cell proliferation. For this, titrated BMDCs were cultured with purified OT-I (A) or OT-II (B) in the presence or absence of the P-gp inhibitor reversin 121 (10 µM) or its vehicle DMSO (0.1%). Cytokine production of IFN-γ (C,D) or TNF-α (E,F) was measured after 48 hr of co-culture. OVA peptide coated BMDCs were fixated with 0.1% PFA for 10 minutes after which OT-I (G) or OT-II (H) proliferation was measured together with IFN-γ (I,J) or TNF-α (K,L) production after 48 hr of co-culture. Representative results are depicted as the mean +/− SEM from three independent experiments performed in triplo. *p<0.05, **p<0.01, ***p<0.001.

To determine the exact role of P-gp during antigen presentation and DC-induced T cell responses, we investigated DC-induced proliferation of ovalbumin-specific CD8^+^ (OTI) and CD4^+^ (OTII) T cells during P-gp blockade. CD4^+^ and CD8^+^ T cell proliferation to OVA-loaded BMDCs was strikingly decreased in the presence of a specific *in vitro* P-gp inhibitor (reversin 121; 200 nM; [24]) compared to its vehicle (Figure 5a,b). Secretion of IFN-γ and TNF-α (Th1 cytokines) was significantly decreased during P-gp inhibited T cell proliferation on different time points (5c–f). Notably, fixation of BMDCs showed no effect of P-gp inhibition on CD4^+^ and CD8^+^ T cell proliferation (Figure 5g,h) or on Th1 cytokine secretion (Figure 5i–l). These results strongly suggest that P-gp on T cells and DCs is required for secretion of inflammatory cytokines like IFN-γ and TNF-α, thereby influencing specific T cell responses. However, as fixation of DCs prevented the P-gp inhibitory effect during T cell proliferation, it is likely that P-gp may exert its dominant role on DCs during immune responses.

### Impaired DC Maturation in Mdr1a/1b −/− Mice

**Figure 6:**
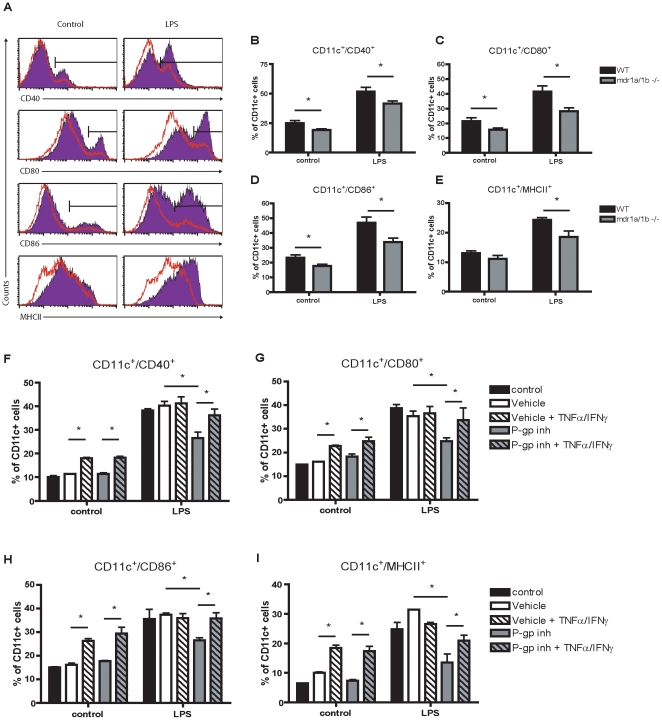
P-gp mediates DC maturation via IFN-γ and TNF-α secretion. (A) DC maturation of BMDCs isolated from WT (shaded histogram) or Mdr1a/1b−/− (open histogram) mice were determined by the expression of CD40, CD80, CD86 or MHCII on live cell gated and CD11c+ cells in the presence or absence of LPS. Quantification of DC maturation of CD11c+ cells positive for CD40 (B), CD80 (C), CD86 (D) and MHCII (E). Recombinant IFN-γ or TNF-α (5 ng/ml) was added to BMDCs derived from C57Bl/6 mice cultured in the presence or absence of LPS, the P-gp inhibitor reversin 121 (10 µM) or its vehicle DMSO (0.1%). Experiments were performed in triplicate using 5 mice per group (A–E) or three independent experiments (F–I) and were presented as the mean +/− SEM. *p<0.05.

During pathogenic immune responses like EAE, DCs undergo maturation upon capture and presentation of antigens to T cells in lymphoid organs and in perivascular spaces surrounding the cerebral vessels [25]. In Mdr1a/1b−/− mice we observed a decreased brain infiltration of T cells and an impaired T cell response during EAE compared to wild-type animals (Figure 1–3). To assess whether this was due to diminished DC maturation, we generated BMDCs from Mdr1a/1b−/− and wild-type mice and stimulated them with lipopolysaccharide (LPS). Unstimulated BMDCs from Mdr1a/1b−/− mice displayed a more immature DC phenotype than wild-type mice (Figure 6a) as determined by a lower level of expression of co-stimulatory molecules CD40, CD80, CD86 and MHCII on CD11c+ DCs (6b–e). Stimulation with LPS resulted in a strongly decreased DC maturation in mdr1a/1b−/− cells compared to wild-type CD11c^+^ DCs (Figure 6a–e). Various cytokines are known to be involved in DC maturation processes and as P-gp may be involved in the cellular extrusion of various cytokines like IFN-γ [14], [16], we therefore hypothesize that the decreased maturation ability of Mdr1a/1b −/− DCs is due to lower efflux of various cytokines during DC maturation. Addition of exogenous TNF-α and IFN-γ together enhanced DC maturation and completely restored the effect of blocking P-gp after LPS addition (Figure 6f–i), whereas addition of these cytokines separately did not affect DC maturation (data not shown). These data strongly suggest that P-gp is crucial in DC function by controlling DC maturation and subsequent T cell activation through cytokine secretion.

## Discussion

Here, we demonstrate a novel *in vivo* physiological role for P-gp as a key regulator of immune responses by controlling DC maturation and subsequent DC-induced T cell responses. We here show that P-gp knockout mice (Mdr1a/1b−/−) displayed reduced clinical signs of EAE compared to wild-type animals. Observed differences were associated with decreased brain inflammation, CNS demyelination and an overall reduced T cell response in Mdr1a/1b−/− animals during disease.

Interestingly, we observed reduced infiltration of CD3^+^ T cells into the brains of Mdr1a/1b−/− EAE lesions, which correlated with diminished clinical signs of EAE and an impaired T cell response in Mdr1a/1b−/− mice compared to wild-type mice. EAE is a T cell–mediated CNS autoimmune disease widely used as an animal model of MS, sharing its clinical, immunological and pathological characteristics [5]. Like MS, EAE is originally thought to be a CD4^+^ T cell mediated disease, although evidence is emerging for a crucial role for CD8^+^ T cells [26]. Notably, lymph node cells from rMOG-immunized Mdr1a/1b−/− mice showed significantly impaired MOG specific T cell proliferation during EAE compared to wild-type animals, whereas a polyclonal stimuli like PMA/ionomycin showed no significant differences (unpublished data). Diminished proliferation coincided with decreased Th1 and Th2, but not Th17 cytokine levels. These results suggest that P-gp is involved in Th1 and Th2 responses during EAE and point to an overall impaired T cell response in Mdr1a/1b−/− animals during EAE.

Reduced clinical signs in Mdr1a/1b −/− mice during EAE may be caused by an altered immune response to myelin proteins in these animals. During the acute and progressive phase of EAE, we observed less brain infiltrating T cells in Mdr1a/1b −/− EAE lesions. These results strongly imply that the ability of Mdr1a/1b −/− T cells to become activated during immune responses is impaired. However, anti-CD3/anti-CD28 stimulation of T cells from Mdr1a/1b −/− displayed a normal activation capacity, indicating that P-gp on T cells is not required for T cell activation responses, which has previously been suggested for Mdr1a encoded P-gp [27]. Interestingly, anti-CD3/anti-CD28 stimulation of Mdr1a/1b −/− lymph node cells resulted in a reduced release of the inflammatory cytokines TNF-α and IFN-γ compared to wild-type cells, whereas the level of intracellular cytokines or transcripts remained unaffected. These results strongly indicate that P-gp mediates the cellular extrusion of inflammatory cytokines from lymph node cells but not its cellular production. As P-gp does not affect the ability of T cells to become activated, the observed differences in T cell responses during EAE may be initiated by P-gp on DCs, as these cells are crucial mediators in regulating T cell responses [7]. P-gp is expressed on both mouse and human T cells and DCs [14], [20], [28] and is upregulated during DC maturation [21]. We here demonstrate that blocking P-gp during DC-induced T cell activation prevented specific CD4^+^ and CD8^+^ T cell proliferation, which was accompanied by reduced secretion of proinflammatory cytokines TNF-α and IFN-γ. Notably, fixation of DCs restored the inhibitory effect of P-gp during CD4^+^ and CD8^+^ T cell proliferation, demonstrating a crucial role for P-gp on DCs for T cell activation via cytokine secretion. P-gp has previously been reported to be involved either directly or indirectly in the extrusion of proinflammatory cytokines like IFN-γ from T cells [16], and IL-12 from DCs [14], [21]. Autocrine IL-12 is necessary for IFN-gamma production by DCs and the production of IL-12 during DC-induced T cell responses can influence DC induction of a Th1 or Th2 immune response [29], which in turn may be responsible for the observed reduced levels of IFN-γ in our assays. However, in our studies levels of IL-12 were too low to detect differences between Mdr1a/1b −/− and wild-type animals (unpublished results). Together, these results imply an important role for P-gp in DC function and subsequent DC-induced T cell responses through regulation of cytokine excretion.

The maturation status of DCs is a key determinant of how the immune response will evolve as it determines specific T cell responses [9]. We here show that DCs that lack P-gp function, either genetically or pharmacologically, have decreased levels of maturation, as determined by the expression of co-stimulatory molecules like CD40, CD80, CD86 and MHC class II antigens on CD11c^+^ DCs. Reduced DC maturation in Mdr1a/1b −/− animals can contribute to the impaired immune response observed *in vivo* during EAE, thereby demonstrating a pivotal role for P-gp as an immunomodulatory molecule. Interestingly, we are the first to show a defective DC phenotype in Mdr1a/1b−/− mice as determined by an impaired DC maturation capacity. We postulate that P-gp mediates DC maturation by influencing the excretion of proinflammatory cytokines like TNF-α and IFN-γ as lower levels of these cytokines were detected during DC-T cell interactions. However, a controversial issue remains whether P-gp itself is capable of transporting cytokines as suggested by some groups [14], [16], [21] or that P-gp is involved in the secretion of other relevant physiological substrates like platelet activating factor [19] that in turn may affect cytokine secretion like IFN-γ [30] as a secondary effect. Nevertheless, addition of TNF-α and IFN-γ to P-gp deficient DCs restored their maturation capacity, highlighting an important role for P-gp and these cytokines during DC maturation and subsequent immune responses.

Together, we here demonstrate an impaired adaptive immunity response in Mdr1a/1b−/− mice *in vivo*, as these mice develop reduced clinical signs of EAE. Reduced clinical signs coincided with decreased levels of DC maturation and impaired DC-induced T cell responses. DCs regulate Th1 and Th2 responses by the production of various cytokines [29], and we hypothesize that P-gp mediates DC maturation and T cell responses by influencing the extrusion of proinflammatory cytokines like TNF-α and IFN-γ, thereby regulating immune responses. Our results indicate a novel *in vivo* physiological role for P-gp during immune processes, which may open new avenues for the treatment of various immune mediated diseases. Based on our results, the prime target for the treatment of MS is affecting P-gp on DCs by immunotherapy, as DCs are ideal tools for immunotherapeutic strategies due to their intrinsic capacity to efficiently present antigens to T cells thereby regulating T cell responses. Interestingly, two compounds, IFN-β and glatiramer acid (GA) that are widely used in the clinic for MS patients have DCs as potential targets [31], [32]. In conclusion, our work highlights a new immunomodulatory role of P-glycoprotein in adaptive immunity and reveals a new target for immunotherapy to suppress adaptive immunity during immune responses and neuroinflammation.

## Materials and Methods

### Mice

Female Mdr1a/1b−/− and wild type (FVB) mice, 8–12-week of age, were kindly provided by dr. Alfred Schinkel (NKI Amsterdam). Mdr1a/1b−/− mice showed no indications for spontaneous colitis after extensive pathological screening and displayed no differences in BBB permeability for non-P-gp substrates [33]. Female C57BL/6-J or C57BL/6-N mice, 7–12 weeks of age, were obtained from Charles River (L'Arbresle, France) or Janvier (France), respectively. OT-I and OT-II mice were bred at the animal facility of the VU University Medical Center (Amsterdam, The Netherlands). OT-I and OT-II mice have transgenic Vα2Vβ5 T cell receptors that recognize the OVA_257–264_ peptide in the context of H2-K^b^ and the OVA_323–339_ peptide in the context of I-A^b^, respectively. All mice were kept under specific pathogen-free conditions and used in accordance with local animal experimentation guidelines.

### Induction of Chronic EAE in Mdr1a/1b and Wild-Type (FVB) Mice

EAE was induced in 8–12-week-old female Mdr1a/1b−/− and wild type (FVB) mice via subcutaneous immunization with 200 µg recombinant myelin oligodendrocyte glycoprotein (rMOG 1–125; synthesized as described [34]) in an emulsion mixed (volume ratio 1∶1) with Complete Freund's Adjuvant (CFA; Difco Laboratories) containing 500 µg of heat-killed *Mycobacterium tuberculosis* H37Ra (MBT; Difco). Control (CFA) animals were injected with saline mixed with CFA containing 500 µg of heat-killed MBT. All animals were additionally intraperitoneally (i.p.) injected with 200 ng pertussis toxin derived from *Bordetella pertussis* (Sigma, Zwijndrecht, The Netherlands) in 200 µL saline at the time of, and after 24 hr following immunization. Mice (n = 23 per group) were examined daily for clinical signs of EAE and were scored as followed: 0, no disease; 1, limp tail; 2, hindlimb weakness; 3, complete hindlimb paralysis; 4, hindlimb paralysis plus forelimb paralysis; and 5, moribund or dead. Mice were killed at day 15 or 29 using O_2_/CO_2_. All experimental procedures were reviewed and approved by the Ethical Committee for Animal Experiments of the VU University Medical Center (Amsterdam, The Netherlands)

### Histology and Immunohistochemistry

Four animals per group were sacrificed for T cell proliferation and histological examination on the peak of disease (day 15) and at the end of disease (day 29). Brains of sacrificed animals were dissected, snap-frozen in liquid nitrogen, and stored at −80°C. Kluver-Barrera (Luxol fast blue/cresyl violet) staining was performed on cryostat sections (5 µm) as described (Breij et al, 2005). For immunohistochemistry, cryostat sections were fixed in ice-cold acetone for 10 minutes and blocked with normal mouse serum (NMS) prior to antibody staining. Immunofluorescence staining was performed in PBS, supplemented with 0.1% (wt/vol) bovine serum albumin (BSA) as previously described [35], [36] and all antibodies are described in Table S1. Monoclonal conjugated antibodies for CD3, CD4 or CD8 were used to detect infiltrated T cells and monocyte infiltration was detected with the conjugated monocyte/macrophage marker mac-1. Cerebral blood vessels were detected with an unconjugated polyclonal antibody directed against laminin and binding was revealed using goat anti-rabbit Alexa Fluor® 546 (Invitrogen). Sections were rinsed, dried, and mounted in Vectamount (Vector Laboratories). Immunofluorescent sections were enclosed in Vinol (Air Products, Allentown, USA) supplemented with DAPI (Invitrogen) and analyzed on a Leica DM6000 fluorescence microscope (Leica Microsystems, Heidelberg, Germany), equipped with LAS AF (Leica) software.

### Immune Cell Activation Assays and Cytokine Analysis

Lymph node cells (1×10^6^ or 0,5×10^6^ cells/well respectively) were isolated as described [37] and were cultured in flat-bottomed, 96-well plates in media (IMDM supplemented with 2 mM L-glutamine, 100 U ml-1 penicillin, 0.1 mg ml-1 streptomycin, 0.5 M 2-mercaptoethanol and 10% fetal calf serum). Cells were subsequently stimulated with rMOG (1–125) in various concentrations (3–30 µg/ml). As a positive control, cells were stimulated with PMA (20 ng/ml; Sigma)/ionomycin (500 ng/ml; Sigma). To assess proliferation rate, cultures were pulsed with [^3^H]-thymidine (1 µCi/well) after 48 h of culture and harvested 18 h later onto filter paper. The counts per minute (c.p.m.) of incorporated [^3^H]-thymidine were measured on a Wallac-LKB Betaplate 1205 liquid scintillation counter. Cytokines were measured in the supernatants of cultured cells using a mouse inflammation CBA kit (BD Pharmingen). Supernatants were taken at four different time points after stimulation (24, 48, 72 and 96 hrs). For lymph node activation assays, cells were pooled from four animals per group and triplicate wells plated.

### In Vitro Ag-Presentation Assays

Bone marrow derived DCs (BMDCs) were isolated and cultured in the presence of GM-CSF as described previously [38]. BMDCs were coated with 1 µg/ml MHC class I-restricted OVA_257–264_ peptide or 10 µg/ml MHC class II-restricted OVA_323–339_ peptide for 30 minutes and washed three times and control or fixated (PFA 0.1%, 10 min) BMDCs used as stimulators for naive T cells in a [^3^H]-thymidine incorporation assay in the presence or absence of the in vitro P-gp inhibitor reversin 121 (10 µM; Alexis) or its vehicle DMSO (0.1%). T cells were obtained from spleen and lymph nodes from OT-I and OT-II transgenic mice. CD8^+^ T cells and CD4^+^ T cells were purified by negative depletion using bead-based T cell isolation kits (Dynal Biotec ASA, Oslo, Norway) from OT-I and OT-II mice, respectively, following manufacturer's protocols. Purity of T cell preparations was between 80 and 90%. After 48 h incubation in a CO_2_ incubator, plates were pulsed with 1 µCi/well of [^3^H]-thymidine. After additional 16 h of incubation, [^3^H]-thymidine incorporation was measured on a Wallac-LKB Betaplate 1205 liquid scintillation counter. Experiments were performed in triplicate and presented as the mean +/− SEM.

### T Cell Activation

Lymph node cells were stimulated with anti-CD3 (10 ug/ml)/anti-CD28 (2 ug/ml) for 5 hr. Cell surface expression of CD44, CD69, CD62L, CD25 was determined by gating on CD4^+^ or CD8^+^ T cells (Table S1). All staining procedures were performed using ice-cold PBS containing 1% BSA and 0.02% Na-azide. Intracellular IFN-γ expression was determined by permeabilization and staining of cells with reagents provided in the Cytofix/Cytoperm kit according to the manufacturer's instructions (BD Pharmingen). Flow cytometry was performed on a FACSCalibur™ with the use of CELLQuest™ software (Becton Dickinson, Franklin Lakes, NJ) with auto-fluorescent cells excluded.

### BMDC Maturation

BMDC were stimulated with or without LPS in the absence or presence of the P-gp inhibitor reversin 121 (10 µM; Alexis) or mouse recombinant cytokines cytokines IFN-γ and TNF-α (5 ng/ml; Peprotech). After 16 hrs, cells were washed with PBS/BSA 0.1% and stained for the expression of co-stimulatory molecules CD40, CD80, CD86 and MHCII on CD11c+ cells (Table S1). Cells were fixed in 2% PFA and flow cytometry was performed as described above.

### RNA Isolation and Real-Time Quantitative PCR

Messenger RNA was isolated from control or stimulated (anti-CD3/anti-CD28 see above) lymph node cells using an mRNA capture kit (Roche) according to the manufacturer's instructions. cDNA was synthesized with the Reverse Transcription System kit (Promega, USA) following manufacturer's guidelines and RT-PCR was performed as described previously [39]. All primer sequences are listed in Table S2 and expression levels of transcripts obtained with real time PCR were normalized to GAPDH expression levels.

### Statistical Analysis

EAE clinical scores for groups of mice are presented as the mean clinical score +/− s.e.m and statistical differences were analyzed by the Mann-Whitney U nonparametric ranking test. All other data were analyzed statistically by means of analysis of variance (ANOVA) and Student-Newman-Keuls test. Statistical significance was defined as *p<0.05, ** p<0.01, *** p<0.001.

## Supporting Information

Figure S1 Decreased demyelination in mdr1a/1b−/− EAE lesions. Brains were isolated from non-immunized control mice (A) or EAE mice 29 days after immunization (B,C) and the cerebellum white matter was analyzed for demyelination using Kluver-Barrera staining in control (A), WT (B) or mdr1a/1b−/− (C) mice. Arrows indicate demyelinated areas and arrowheads indicate infiltrated leukocytes. Images represent representative tissues from 4 mice per group. Magnification 10x.
(0.90 MB TIF)

Table S1 (0.02 MB DOC)

Table S2 (0.02 MB DOC)

Table S3 (0.02 MB DOC)
