# Correction: P-Glycoprotein Acts as an Immunomodulator during Neuroinflammation

**DOI:** 10.1371/annotation/1320f032-3c02-4feb-94ec-66591aa27d1c

**Published:** 2010-01-22

**Authors:** Gijs Kooij, Ronald Backer, Jasper J. Koning, Arie Reijerkerk, Jack van Horssen, Susanne M. A. van der Pol, Joost Drexhage, Alfred H. Schinkel, Christine D. Dijkstra, Joke M. M. den Haan, Teunis B. H. Geijtenbeek, Helga E. de Vries

The eighth author's name appears incorrectly. The correct name is: Alfred H. Schinkel. 

