# Supplementary figures and images for: P-Glycoprotein Acts as an Immunomodulator during Neuroinflammation
Source: PLoS One. 2009 Dec 8;4(12):e8212. doi: 10.1371/journal.pone.0008212 (PMC2785479; doi:10.1371/journal.pone.0008212)

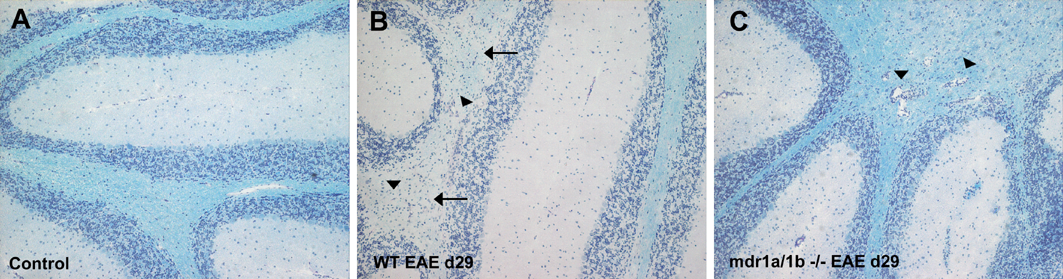

Supplement: Figure S1 — Decreased demyelination in mdr1a/1b−/− EAE lesions. Brains were isolated from non-immunized control mice (A) or EAE mice 29 days after immunization (B,C) and the cerebellum white matter was analyzed for demyelination using Kluver-Barrera staining in control (A), WT (B) or mdr1a/1b−/− (C) mice. Arrows indicate demyelinated areas and arrowheads indicate infiltrated leukocytes. Images represent representative tissues from 4 mice per group. Magnification 10x. (0.90 MB TIF) [file pone.0008212.s001.tif]
