# Supplementary material for: P-Glycoprotein Acts as an Immunomodulator during Neuroinflammation
Source: PLoS One. 2009 Dec 8;4(12):e8212. doi: 10.1371/journal.pone.0008212 (PMC2785479; doi:10.1371/journal.pone.0008212)
Supplement: Table S1 — (0.02 MB DOC) [file pone.0008212.s002.doc]

| **Supplementary table 1. Antibodies** | | | |
| --- | --- | --- | --- |
| Primary antibody | Clone | Company |  |
|  |  |  |  |
| CD3 | KT.3 | Molecular Cell Biology and Immunology, Amsterdam, The Netherlands |  |
| CD4  CD8 | GK1.5  53-6.7 | eBiosciences, San Diego, USA  eBiosciences, San Diego, USA |  |
| CD11b (Mac-1)  CD11c  CD25  CD28  CD40  CD44  CD62L  CD69  CD80  CD86  IFN-γ  Laminin (pAb)  MHCII | M1/70  N418  PC61  37.51  1C10  IM781  MEL-14  H1.2.F3  16-10A1  GL1  XMG1.2  -  M5/114 | eBiosciences, San Diego, USA  eBiosciences, San Diego, USA  eBiosciences, San Diego, USA  eBiosciences, San Diego, USA  eBiosciences, San Diego, USA  Molecular Cell Biology and Immunology, Amsterdam, The Netherlands  BD Pharmingen, San Jose, USA  BD Pharmingen, San Jose, USA  eBiosciences, San Diego, USA  eBiosciences, San Diego, USA  eBiosciences, San Diego, USA  MP Biomedicals, Illkirch, France  Molecular Cell Biology and Immunology, Amsterdam, The Netherlands |  |
