# Supplementary material for: P-Glycoprotein Acts as an Immunomodulator during Neuroinflammation
Source: PLoS One. 2009 Dec 8;4(12):e8212. doi: 10.1371/journal.pone.0008212 (PMC2785479; doi:10.1371/journal.pone.0008212)
Supplement: Table S2 — (0.02 MB DOC) [file pone.0008212.s003.doc]

| **Supplementary table 2. Primer sequences used for RT-PCR** |
| --- |
| **Gene** **Forward primer (5-3’) Reverse primer (5-3’)** |
| GAPDH CCATGTTCGTCATGGGTGTG GGTGCTAAGCAGTTGGTGGTG  TNF-α CCGTCAGCCGATTTGCTATC TGACGGCAGAGAGGAGGTTG  IFN-γ CAGCAACAGCAAGGCGAAA AGCTCATTGAATGCTTGGCG |
