# Supplementary material for: P-Glycoprotein Acts as an Immunomodulator during Neuroinflammation
Source: PLoS One. 2009 Dec 8;4(12):e8212. doi: 10.1371/journal.pone.0008212 (PMC2785479; doi:10.1371/journal.pone.0008212)
Supplement: Table S3 — (0.02 MB DOC) [file pone.0008212.s004.doc]

| **Supplementary table 3. Immune cell quantification in EAE lesions** | | | |
| --- | --- | --- | --- |
| Mice Cells | Nr. of cells | % perivascular % parenchyma |  |
|  |  |  |  |
| **Wild-type** CD3^+^ T cells  **day 15 EAE** Macrophages | 429 +/- 13  405 +/- 21 | 46.2 +/- 3.2 53.8 +/- 2.8  47.8 +/- 7.1 52.2 +/- 6.5 |  |
| **Mdr1a/1b-/-** CD3^+^ T cells  **day 15 EAE** Macrophages | 285 +/- 11*  211 +/- 10* | 76.1 +/- 4.3 23.9 +/- 3.7  67.6 +/- 2.4 32.4 +/- 3.8 |  |
| **Wild-type** CD3^+^ T cells  **day 29 EAE** Macrophages  **Mdr1a/1b-/-** CD3^+^ T cells  **day 29 EAE** Macrophages | 377 +/- 20  354 +/- 13  154 +/- 9*  180 +/- 11* | 41.9 +/- 4.6 58.1 +/- 2.9  38.5 +/- 3.4 61.5 +/- 5.1  80.1 +/- 3.2 20.9 +/- 2.2  69.1 +/- 2.4 29.9 +/- 3.5 |  |
